# A Link between Autophagy and the Pathophysiology of LRRK2 in Parkinson's Disease

**DOI:** 10.1155/2012/324521

**Published:** 2012-12-03

**Authors:** Patricia Gómez-Suaga, Elena Fdez, Marian Blanca Ramírez, Sabine Hilfiker

**Affiliations:** Instituto de Parasitología y Biomedicina (López-Neyra), Consejo Superior de Investigaciones Cientificas (CSIC), Granada, 18100 Armilla, Spain

## Abstract

Parkinson's disease is a debilitating neurodegenerative disorder, and its molecular etiopathogenesis remains poorly understood. The discovery of monogenic forms has significantly advanced our understanding of the molecular mechanisms underlying PD, as it allows generation of cellular and animal models carrying the mutant gene to define pathological pathways. Mutations in leucine-rich repeat kinase 2 (LRRK2) cause dominantly inherited PD, and variations increase risk, indicating that LRRK2 is an important player in both genetic and sporadic forms of the disease. G2019S, the most prominent pathogenic mutation, maps to the kinase domain and enhances enzymatic activity of LRRK2, which in turn seems to correlate with cytotoxicity. Since kinases are druggable targets, this has raised great hopes that disease-modifying therapies may be developed around modifying LRRK2 enzymatic activity. Apart from cytotoxicity, changes in autophagy have been consistently reported in the context of G2019S mutant LRRK2. Here, we will discuss current knowledge about mechanism(s) by which mutant LRRK2 may regulate autophagy, which highlights additional putative therapeutic targets.

## 1. Introduction

Parkinson's disease (PD) is a common neurodegenerative disorder with symptoms including tremor, rigidity, and postural instability [1]. Autosomal-dominant mutations in leucine-rich repeat kinase 2 (LRRK2) comprise the most common monogenic form of PD [2–5]. LRRK2-associated PD is symptomatically and neurochemically largely indistinguishable from sporadic PD cases [6], even though the reported pleomorphic pathology of mutant LRRK2 carriers differs from the rather classical *α*-synuclein pathology associated with sporadic PD. Variations in LRRK2 have further been reported to increase risk for sporadic PD [7–9], which implicates LRRK2 in both sporadic and familial forms of the disease. The big advantage of studying the function of a mutated gene product as compared to a sporadic disease is that one can generate cellular and animal models carrying the mutant gene to define pathological pathways. In conjunction with the described enzymatic activity of LRRK2 which may be targeted by select kinase inhibitors [10, 11], this has propelled the protein into the limelight of PD research worldwide. However, to develop disease-modifying or neuroprotective therapies around LRRK2, a clear understanding of its normal and pathological function(s) is required. A link between LRRK2 and aberrant macroautophagy has been consistently observed, and here we review our current knowledge of LRRK2's role in autophagy and lysosomal homeostasis with implications for cell demise in PD.

## 2. LRRK2 Structure and Cellular Localization

**Figure 1:**
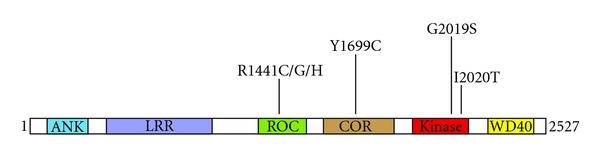
Domain structure and PD mutations of LRRK2. The central region of LRRK2 contains a GTPase domain also called (ROC), a C-terminal of ROC (COR) domain of unknown function, and a kinase domain, flanked on either side by protein-protein interaction domains including an ankyrin repeat domain (ANK), leucine-rich repeats (LRR) and a WD40 domain (WD40). Clearly causative pathogenic mutations are indicated and are clustered around the catalytic domains of LRRK2. Only the G2019S mutation consistently augments kinase activity.

LRRK2 is a large multidomain protein belonging to the ROCO family of proteins which are characterized by the presence of leucine-rich repeats, a Ras of complex (ROC) GTPase domain, a C terminal of ROC (COR) linker region, and a kinase domain [12]. Among the many putative pathogenic variants identified to date, six missense mutations in LRRK2 have been clearly shown to segregate with disease, and thus represent authentic disease-causing variants [13]. Importantly, these mutations all map to the central region comprised of the catalytic domains, indicating that a change in enzymatic activity (either GTPase or kinase) mediates the pathogenic effect(s) of LRRK2 (Figure 1). The G2019S mutation within the kinase domain (Figure 1) is the most frequent pathogenic LRRK2 mutation, having been identified in up to about 40% of familial PD cases dependent on ethnicity, and also detected in apparent sporadic PD cases [4, 5, 7–9]. This mutation has been consistently shown to augment catalytic activity [14], even though the inherent kinase activity of LRRK2 is very low. This may be, at least in part, due to the lack of currently identified and reproducible genuine kinase substrates. LRRK2 kinase is active towards itself [14], and autophosphorylation may represent a physiological readout. The effect of other pathogenic mutations on kinase activity is less clear. Intriguingly, a recent study indicates that the G2385R risk variant causes a partial loss of kinase activity, highlighting the possibility that both too much or too little LRRK2 kinase activity may be detrimental [15]. Mutations in the ROC and COR domain cause a decrease in GTPase, without gross changes in kinase activity [16, 17], suggesting that the GTPase activity may comprise the genuine physiological readout of LRRK2, which may be further modulated by kinase activity [11]. Finally, apart from the catalytic central domains, LRRK2 contains various protein-protein interaction domains including LRRK2-specific, ankyrin, and leucine-rich repeat motifs at the N-terminus, and WD40 repeats near the C-terminus of the protein (Figure 1). The existence of these domains indicates the possibility that it may act as a protein scaffold for the assembly of protein complexes [18]. Indeed, LRRK2 has been reported to interact with a whole array of proteins and may form distinct protein complexes in a cell-type or subcellular compartment-specific manner [19]. In this context, the enzymatic activities of LRRK2 may serve to change the affinity and/or composition of such complexes. Alternatively, a change in enzymatic activity may be the result of a change in protein complex interaction(s). Consistent with the latter possibility, LRRK2 has been reported to exist as a dimer, with dimerization enhancing kinase activity and causing relocalization to intracellular membranes [20–23], even though this has been disputed [24]. In either case, apart from being cytosolic, overexpressed, as well as endogenous, LRRK2 has been reported to localize to specific membrane subdomains including endolysosomal structures in neuronal and non-neuronal cells [25–27]. There, it may interact with and/or regulate distinct protein complexes. Such interactions may be controlled by the catalytic activity of LRRK2, either towards itself or currently unknown substrates. If correct, not only the catalytic activity of LRRK2, but also the modulation of distinct protein interactions should be considered possible targets for therapeutic strategies.

## 3. LRRK2 and the Regulation of Autophagy

The precise molecular mechanism(s) of LRRK2 function remain unclear. Certain phenotypes are robustly seen, such as the acutely toxic nature of pathogenic mutant forms of LRRK2 upon high-level overexpression in cultured cells [28–31]. Cell death is also evident upon viral vector-mediated expression of mutant LRRK2 *in vivo* [32, 33], and toxicity seems to depend on kinase activity [28, 29, 32]. In neuronal cellular models where cell death is not apparent, neurite shortening represents another consistent phenotype associated with mutant LRRK2 expression [34–42]. Where investigated, this also seems kinase activity-dependent and mediated by macroautophagy [34, 35, 41, 42]. All mutations tested to date have at least one of these effects on cells. Thus, the cellular pathway(s) underlying LRRK2 toxicity may involve altered macroautophagy, which in neurons may lead to neurite shortening and eventual cell demise. If so, elucidating the mechanism(s) by which LRRK2 alters macroautophagy becomes key.

Apart from playing an important role in determining neurite length [43], macroautophagy (thereafter named autophagy) has recently gained attention for its contribution to the pathogenesis of several neurodegenerative diseases including PD [44–46]. Autophagy is a process by which cytosolic constituents, including damaged organelles and aggregated proteins, are engulfed within specialized double-membraned vesicles called autophagosomes. Autophagosomes then fuse with amphisomes or lysosomes, followed by the hydrolytic degradation of products in lysosomes and reformation of these organelles to maintain cellular degradative capacity [47, 48]. Disrupting any part of this process impairs autophagic flux, accompanied by the accumulation of autophagic substrates and organelles [47, 48]. In addition, autophagy and endocytosis share lysosomes as their common end-point [49], such that it has been very difficult to define whether LRRK2 plays positive or negative roles in autophagic-lysosomal clearance. 

A wealth of studies indicate that LRRK2 regulates autophagy. For example, various lines of knockout mice have been generated, which display an increase in the number and size of secondary lysosomes and autolysosome-like structures in the kidney [50–52]. An accumulation of lipofuscin granules, highly oxidized, and crosslinked proteins and lipids which cannot be properly degraded, and p62, an autophagy substrate, have also been observed [50–52]. Such abnormal accumulation of undigested material indicates an impairment in the autophagosomal-lysosomal degradation system. To determine a possible defect along the autophagic pathway, the levels of LC3I and LC3II have been analyzed. LC3II, the lipidated form of LC3I, becomes bound to the autophagosomal membrane and serves as a reliable indicator of autophagic activity [53]. Studies analyzing the levels of LC3II in the absence of LRRK2 in the kidney indicate either no change [52], or a biphasic change with an initial enhancement of flux at young age, followed by an impairment of flux over time [50, 51]. This block in flux has been interpreted to be due to an “overload” of the system, resulting in impaired clearance and/or recycling of autophagic components/autolysosomes [51]. Whilst an interesting hypothesis, it depends on assigning a rate-limiting step in the autophagy process, which will need further proof.

In agreement with the *in vivo* data of young animals, RNAi-mediated knockdown of LRRK2 has been found to result in increased autophagic flux under starvation conditions in a human embryonic kidney cell line (HEK293) [25]. Unfortunately, flux experiments were not performed under nutrient-rich conditions in these knockdown cells. Conversely, overexpression of R1441C mutant LRRK2 caused a block in autophagic flux, as evidenced by the accumulation of multivesicular bodies and large autophagosomes containing incompletely degraded material and increased levels of p62 [25]. Similarly, in our studies overexpressing wildtype and G2019S mutant LRRK2 in HEK293 cells, we found improper autophagic-lysosomal clearance, as indicated by an accumulation of autophagic structures and lipid droplets [54, 55]. Thus, at least in the kidney and in kidney-derived cell lines, the normal function of LRRK2 may be related to negatively regulating autophagic clearance and/or lysosomal homeostasis. Too much LRRK2 activity then would dampen, whilst too little activity would enhance autophagic flux. If the latter overloads the system with time, any deregulation of LRRK2 activity may be damaging to the proper functioning of the autophagic pathway *in vivo*.

## 4. Tissue-Specific versus Universal Regulation of Autophagy

 In contrast to kidney, there has been no evidence for the accumulation of autophagic or lysosome-related structures in the brains of aged mice lacking LRRK2 [50–52]. Thus, LRRK2 may perform distinct roles in a tissue-specific manner, with an effect on autophagy in kidney, but not in brain. Alternatively, LRRK1 may functionally compensate for the loss of LRRK2 in the brain, but not in the kidney, the latter of which contains small amounts of LRRK1 versus LRRK2 and thus percentually suffers a much bigger loss of LRRK proteins [56, 57]. In addition, the homo- and heterodimerization of LRRK1 and LRRK2 proteins has been reported [58, 59], with LRRK1 involved in regulating endosomal trafficking [60, 61], consistent with a role for both proteins in recycling and degradation events. Generation of double-knockout lines will be required to delineate whether a complete loss of LRRK proteins in neurons results in age-related changes in autophagy similar to those observed in the kidney.

 As another possibility, the overall levels of LRRK proteins present in different tissues may predetermine whether a phenotype is observed upon knockout versus overexpression conditions. For example, as LRRK levels are very high in kidney [56, 57], a knockout strategy may be more adequate to uncover the (normal) role of LRRK2 in autophagic-lysosomal clearance. Conversely, given the low levels of LRRK2 in the brain, an overexpression approach, especially of mutant, hyperactive LRRK2, may be more effective.

 Apart from differences in the levels of LRRK proteins, the rate of basal autophagy also displays large differences across distinct tissues. Thus, the same pathogenic mutation of LRRK2 may give rise to different degrees of pathology depending on the cellular milieu in which it is operating [19]. As basal autophagy is very high in the kidney, a deregulation may be more pronounced in this organ as compared to other tissues. Nevertheless, if LRRK2 is a universal modulator of autophagic/lysosomal clearance, changes should also be detectable in other tissues such as brain, albeit possibly to a lesser degree or in an age-dependent manner difficult to track using rodent models. In agreement with a universal role in regulating autophagy, an overexpression approach using G2019S mutant LRRK2 has been reported to cause abnormal accumulation of autophagic and lysosomal structures in primary cortical neurons and neuronal cell lines in culture [34, 35]. Similarly, an accumulation of autophagic vacuoles, including early and late autophagosomes, has been described in the soma and processes in the cortex and striatum from G2019S, and to a lesser degree R1441C, transgenic mice with advanced age [40]. Thus, both *in vitro* and *in vivo*, overexpression of mutant LRRK2 seems to cause impaired autophagic-lysosomal clearance in neurons as well. A decrease in autophagic flux, concomitant with an increase in p62 levels, autophagosomes and lipid droplets has recently also been described in human dopaminergic neurons derived from induced pluripotent stem cells from G2019S mutant LRRK2, but not control patients, after long-term culture [42]. These data are important, as they indicate that endogenous levels of mutant LRRK2 are sufficient to induce an autophagic-lysosomal phenotype in dopaminergic neurons with time. In contrast, fibroblasts from those same patients do not reveal differences in autophagic clearance, consistent with their extremely low levels of basal autophagic activity [42]. However, the latter findings are in contrast to a recent report suggesting elevated levels of autophagic activity [62], and the precise role for mutant LRRK2 in autophagy regulation in fibroblasts remains to be determined. Finally, bone marrow-derived macrophages from mutant LRRK2 mice display a decrease in LC3II levels, possibly highlighting an autophagic phenotype in those cells as well [63]. Alltogether, the currently available data indicate that LRRK2 can regulate autophagic-lysosomal clearance in neurons as well as a variety of other cell types, possibly in a manner dependent on the basal level of autophagy.

## 5. Mechanism of Autophagy Regulation by LRRK2

 If LRRK2 indeed regulates autophagic clearance, understanding the mechanism of action becomes important to develop alternative and/or complementary treatment strategies. The effects of LRRK2 on autophagic-lysosomal clearance may reflect its primary mechanism of action or may occur secondarily, elicited as a response to some upstream event(s). Even if direct, many distinct scenarios remain possible, as autophagy intersects with both secretory and endocytic pathways at several points [64]. Given its heterodimerization with LRRK1 [58, 59], which has been reported to regulate trafficking events of the epidermal growth factor receptor (EGFR) between early and late endosomes, endosome motility and sorting of the epidermal growth factor receptor (EGFR) to the inner vesicles of multivesicular bodies [60, 61], one may speculate that LRRK2 regulates similar events, with consequences for autophagic pathways involving multivesicular bodies [65].

 Apart from this mere analogy, LRRK2 has been shown to interact with the GTPase rab5b, a key regulator of early endocytic vesicle trafficking [66]. Overexpression or knockdown of LRRK2 cause a decrease in presynaptic vesicle endocytosis rates, again indicating that both too much and too little LRRK2 adversely alter the balance of homeostatic mechanisms, in this case controlling endocytosis [66]. Similarly, both overexpression or knockdown of LRRK2 induce defects in vesicle endocytosis upon depolarization of primary neuronal cultures [67, 68], which may involve interactions of LRRK2 with a series of endocytic proteins apart from rab5b [68], but further studies are needed to determine how LRRK2 may regulate the function of any of these proteins. Interestingly, rab5b, apart from regulating the endocytic pathway [69] has recently been shown to play an additional positive role in autophagy by regulating an early step of autophagosome formation in a TORC1-independent manner [70]. Thus, a LRRK2-mediated regulation of rab5b may rather directly impact upon autophagic flux. Indirect LRRK2-mediated regulation of autophagy via changes in endocytosis can be envisioned as well, as endocytosis enables the formation of distinct signal transduction complexes which define specialized endosomal-lysosomal signaling platforms [71]. LRRK2-mediated changes in endocytosis may modulate the formation of those intracellular complexes to regulate signalling cascades including Wnt or MAP kinase cascades [71], both of which have been shown to be affected by LRRK2 [18], and which then may modulate the function of downstream autophagic components.

**Figure 2:**
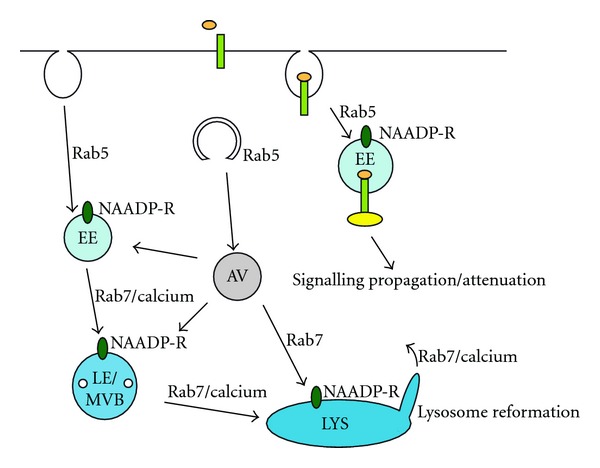
Possible mechanisms by which LRRK2 may regulate events related to endolysosomal and autophagic function. Modulation of rab5 function could cause changes in endocytosis and/or autophagosome formation. Altered endocytosis could also modulate signalling events occurring at the plasma membrane or on intracellular organelles, thereby, indirectly impacting upon autophagy through phosphorylation events of distinct proteins required for the process. At later stages, through modulating rab7 function, LRRK2 may alter the fusion of autophagosomes/endosomes with lysosomes or impair lysosome reformation, which would impact upon autophagic-lysosomal clearance in both cases. As most of the abovementioned membrane fusion/reformation steps require intraluminal calcium, LRRK2 may further regulate endolysosomal clearance by modulating NAADP-sensitive calcium channels (NAADP-R) located on endosomes and lysosomes. The increasing intraluminal calcium concentrations along the endocytic/lysosomal pathway are indicated by the progressively darkened blue color. Ligand binding to receptors, followed by endocytosis and interaction with signalling complexes are schematically indicated. EE: early endosome; AV: autophagosome; LE/MVB: late endosome/multivesicular body; LYS: lysosome. For further details and references, see text.

 Multiple data support the idea that LRRK2 also modulates late steps in the autophagic-lysosomal clearance pathway. The fusion of both autophagosomes and endosomes with lysosomes requires rab7, as does the process of lysosome reformation [49, 72–74], and interfering with rab7 function will thus affect autophagic-lysosomal clearance. Indeed, at least in Drosophila, the LRRK2 homolog seems to interact with rab7 on late endosomes and lysosomes to negatively regulate rab7-dependent perinuclear lysosomal positioning required for the efficient degradation of autophagosomes [75]. Another recent study in C. elegans expressing human wildtype or mutant LRRK2 in conjunction with proteostatic stress indicates increased expression of numerous proteins including a subunit of the V-type proton ATPase [76, 77], and the behavioural motor deficits observed in these double-transgenic worms can be reverted by increasing autophagic flux using a rapamycin analog. These data are consistent with our findings that mutant LRRK2 may increase lysosomal pH and concomitantly decrease lysosomal clearance, a process reverted by rapamycin, but not by other compounds which increase autophagy in an mTOR-independent manner [54]. It remains to be seen whether the beneficial effect of the rapamycin analog on motor output is related to an mTOR-dependent increase in degradative capacity as autophagic flux is enhanced, a decrease in protein synthesis, an effect on lysosomal homeostasis, or a combination thereof. Taken alltogether, a picture is emerging whereby LRRK2 may regulate both early and late steps of autophagic-lysosomal clearance in a rab protein-dependent manner (Figure 2).

## 6. A Link between LRRK2, Autophagy, and NAADP-Mediated Endolysosomal Calcium Signaling

 In agreement with other reports, we also found an increase in autophagosome numbers upon transient overexpression of wildtype and G2019S-mutant, but not kinase-dead LRRK2 in various cell lines including dopaminergic neuroendocrine cells [54, 55]. Interestingly, we found that these effects were inhibited by the calcium chelator BAPTA, suggesting that they were calcium-dependent. The effects of LRRK2 overexpression on autophagosome numbers were also blocked when genetically depleting ER calcium stores and were accompanied by an increase in the pH of a population of lysosomes and an increase in the number of lipid droplets. This phenotype closely matches the one triggered by NAADP, which evokes cytosolic calcium signals that can be amplified by ER calcium stores, causes partial alkalinization of acidic stores, and induces lipid accumulation [78–80]. NAADP is a potent agonist-generated second messenger and capable of triggering complex calcium signals which are initiated from acidic stores and are being subsequently amplified by ER calcium release channels [81–83]. Targets for NAADP are likely comprised of the endolysosomal two-pore channels TPC1 and TPC2 [84–86], even though recent studies indicate that NAADP does not directly bind to TPCs, but rather indirectly through currently unidentified associated low-molecular weight binding proteins [87]. In either case, there is a growing appreciation of the importance of endolysosomal organelles as mobilizable calcium stores [81, 88], and intraluminal calcium seems required for endolysosomal membrane fusion events, thus, directly impacting upon endosomal and autophagic trafficking events [73].

**Figure 3:**
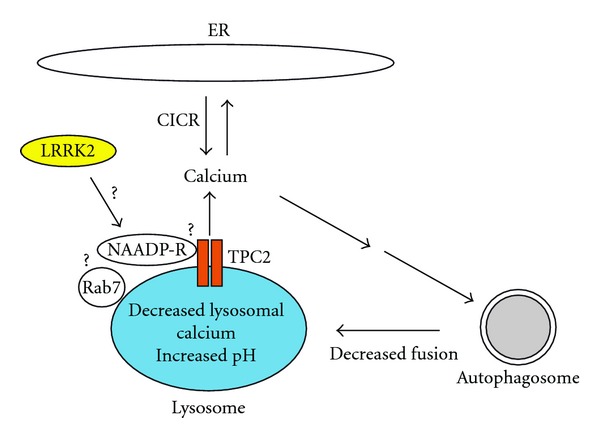
Diagram of proposed mechanism(s) by which LRRK2 regulates autophagy via modulation of NAADP-dependent calcium channels (NAADP-R) on lysosomes. LRRK2 localizes to lysosomes and regulates calcium release through two-pore channels (TPCs). Whether this is due to a direct interaction of LRRK2 with NAADP-R, an indirect interaction via rab7 or additional proteins, or whether it is mediated by a phosphorylation event remains to be determined. Calcium release from acidic organelles then causes calcium-induced calcium release (CICR) from the ER to amplify cytosolic calcium signals, which leads to the activation of a cascade to increase autophagosome numbers. Diminished luminal calcium will further cause a decrease in autophagosome-lysosome fusion, and increased pH may have additional effects on eventually impairing lysosomal proteolysis, leading to the observed autophagic-lysosomal clearance phenotype.

 The analogy between the effects of LRRK2 overexpression and NAADP action prompted us to test the connection between NAADP and LRRK2 action. Accordingly, we found that elevation of cellular NAADP levels using a cell permeable NAADP analogue (NAADP-AM) [89] increases autophagosome numbers, lysosomal pH, and lipid droplet numbers, thus, largely mimicking the effects observed upon LRRK2 overexpression [54]. Conversely, the NAADP antagonist NED19 recently identified by virtual screening methods [90] reverted the effects of LRRK2. The increase in autophagosome number could also be blocked by overexpression of TPC2 mutated within the pore region [91]. This inactive mutant likely acts in a dominant manner similar to TPC1 in which the corresponding residue is mutated [84, 92]. Together, these data uncover a hitherto unknown link between NAADP and LRRK2 function (Figure 3).

## 7. Summary

A wealth of recent data supports the idea that LRRK2 regulates autophagy. Another ROCO protein family member, death-associated protein kinase 1 (DAPK1), also seems to be an essential regulator of autophagy [93], and it will be interesting to determine whether other ROCO proteins are autophagy modulators as well. Furthermore, LRRK2 variants have been associated with Crohn's disease (CD), an inflammatory bowel disease [94]. As other CD-associated risk genes are also linked to autophagy triggered as an antibacterial response, the disease may result from ineffective control of bacterial infection and resultant chronic inflammation [95]. Similarly, recent data suggest that LRRK2 dysfunction in PD may involve the immune system [96], and the involvement of aberrant autophagy in such process warrants further investigation. Whilst the link between LRRK2 and autophagy is becoming solid, the precise underlying mechanism(s) remain unknown. Both direct and indirect scenarios can be envisioned, and evidence for both is emerging. Rab proteins and calcium seem to play potentially important and not mutually exclusive roles. Calcium is known to both positively and negatively regulate autophagy, and these dual effects may depend on the precise intraorganellar location at which it is required for autophagosome-lysosome or endosome-lysosome fusion, respectively [49, 72]. Many questions remain to be addressed, such as whether TPCs (or NAADP binding proteins) are LRRK2 targets, whether LRRK2 causes indeed measurable changes in intracellular calcium levels, or how LRRK2 regulates the activity or localization of distinct rab proteins. Additional work is needed toward delineating the precise molecular links between LRRK2, autophagy, and NAADP-mediated events.
